# Existence and uniqueness of fractal-fractional equations generated by a new fractal-fractional operator utilizing the advanced gamma function

**DOI:** 10.1016/j.mex.2024.102684

**Published:** 2024-03-28

**Authors:** Ibtehal Alazman, Rabha W. Ibrahim

**Affiliations:** a Department of Mathematics and Statistics, College of Science, Imam Mohammad Ibn Saud Islamic University (IMSIU), Riyadh, Saudi Arabia; b Department of Mathematics, Near East Boulevard, Mathematics Research Center, Near East University, PC: 99138, Mersin, Nicosia 10, Turkey; c Information and Communication Technology Research Group, Scientific Research Center, Al-Ayen University, Thi-Qar, Iraq

**Keywords:** Fractional calculus, Fractal calculus, Fractal-fractional differential operator, Fractal-fractional calculus, Fractal-fractional differential equation, The Rabotnov function

## Abstract

Numerous special functions, including the beta function, hypergeometric functions, and other orthogonal polynomials, are closely connected to the gamma function. Recently, gamma function has been enhanced by adding a new parameter. As a consequence, this gamma is called the parametric gamma function or b-gamma function. By utilizing the enhanced gamma function (or the parametric gamma function), we have present a generalization for the special function Rabotnov function. Consequently, new fractal-fractional operators (derivative and integral) involving the generalized Rabotnov function are defined. Analysis is introduced to discover the main properties of the suggested operators. Since the main challenge in the calculus of fractal-fractional operators is to design examples, we illustrate a set of examples including power series. We explore the boundedness of the recommended operators. There are many gains for checking the boundedness of fractal operators in general and fractal-fractional operators in particular. Moreover, as an application, we establish the existence and uniqueness solution of abstract fractal-fractional equation. Examples are presented at the end of the effort. Graphics and computations are observed using MATHEMATICA 13.3 software.

• By using the parametric gamma function, the Rabotnov special function is generalized. New fractal-fractional operators are presented using the generalized Rabotnov function with examples;
• Boundedness of these operators is investigated, where bounded operators give a strong foundation for understanding linear transformations across normed spaces, with many applications in science and mathematics.
• Conditions of the existence and uniqueness of solutions of fractal-fractional differential abstract equation are established.

## Graphical abstract

**Figure:**
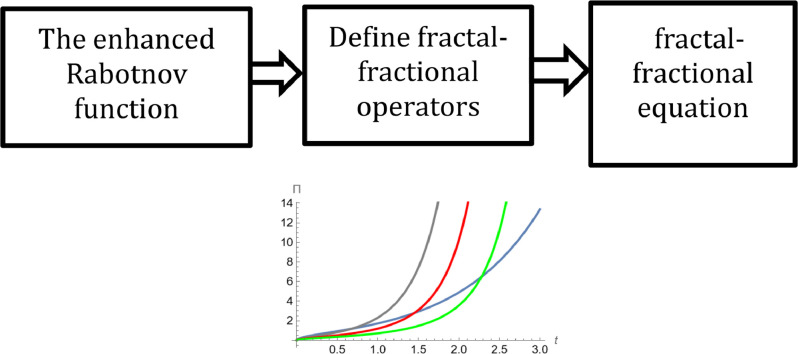

Specifications table

**Table:** 

Subject area:	Mathematics and Statistics
More specific subject area:	*Fractal-Fractional Calculus*
Name of your method:	*The Rabotnov function*
Name and reference of original method:	*The name of the method is: Rabotnov Function* *Reference:* *Rabotnov, Yury. “Equilibrium of an elastic medium with after-effect.” Fractional Calculus and Applied Analysis 17, no. 3 (2014): 684–696. DOI:*10.2478/s13540-014-0185-1
Resource availability:	*N/A*

## Method details

### Background

A mathematical function that extends the factorial function to complex numbers is the gamma function, represented by the symbolΓ(.). With the exception of non-positive integers, for which it has simple poles, it can be used for all complex numbers. Numerous areas of mathematics, such as information, number theory, and complex analysis, rely heavily on the gamma function. The generalized gamma function can be engaged in the concept of fractal-fractional calculus to generalize the differential and integral operators. These operators have complex, self-similar properties and aid in the description of events with non-integer scaling. In addition, gamma functions is involved to describe the structure of a general class of special functions, like Mittag-Leffler function and its generalizations (see [1], [2], [3]).

Special functions are mathematical functions with particular characteristics or uses that are often employed in science, engineering, mathematics, as well as other technical domains. These functions are more complex than trigonometric, exponential, and polynomial functions. These unique functions frequently show up as treatments for differential equations that come up in a variety of fields including engineering and scientific challenges. They have been thoroughly examined and analyzed for practical use, and they offer an improved succinct and effective means of expressing certain mathematical connections (see [4], [5], [6]).

We have introduced new fractal-fractional operators (integral and derivative) incorporating Rabotnov function by using the parametric gamma function. In order to identify the primary characteristics of the proposed operators, analysis is presented. Since creating samples is the primary difficulty in the calculus of fractal-fractional operators, we provide a collection of illustrations, including power series. Furthermore, we prove that the abstract fractal-fractional equation has a unique solution and exists. At the conclusion of the endeavor, applications are given out.

#### Concepts

The domain of the gamma function is expanded to embrace a parameter that describes the shape b by the enlarged gamma function. Benefits of the modified gamma function may be found in many fields of mathematics, physics, and electronics; in specifically, it is useful in issues related to statistical distributions, probability theory, and complex analysis. This section deals with all concepts that will be used in the sequel. The b−gamma function of the motivation, as well as commonly known as the generalized gamma function, can be seen, as follows [7]

$$\Gamma_{b}\left(t\right)=\underset{m\to \infty }{\text{lim}}\frac{m!b^{m}{\left(m\;b\right)}^{\frac{t}{b}-1}}{{\left(t\right)}_{m,b}},b>0$$

$${\left(t\right)}_{m,b}≔\frac{\Gamma_{b}\left(t+m\;b\right)}{\Gamma_{b}\left(t\right)}.$$

And also satisfies the following properties (see [7])

• $\Gamma \left(t\right)=\text{li}m_{b\to 1}\Gamma_{b}\left(t\right);$
• $\Gamma_{b}\left(t\right)=b^{\frac{t}{b}-1}\Gamma \left(\frac{t}{b}\right);$
• $\Gamma_{b}\left(t+b\right)=t\Gamma_{b}\left(t\right);$
• $\Gamma_{b}\left(b\right)=1.$

**Fig. 1:**
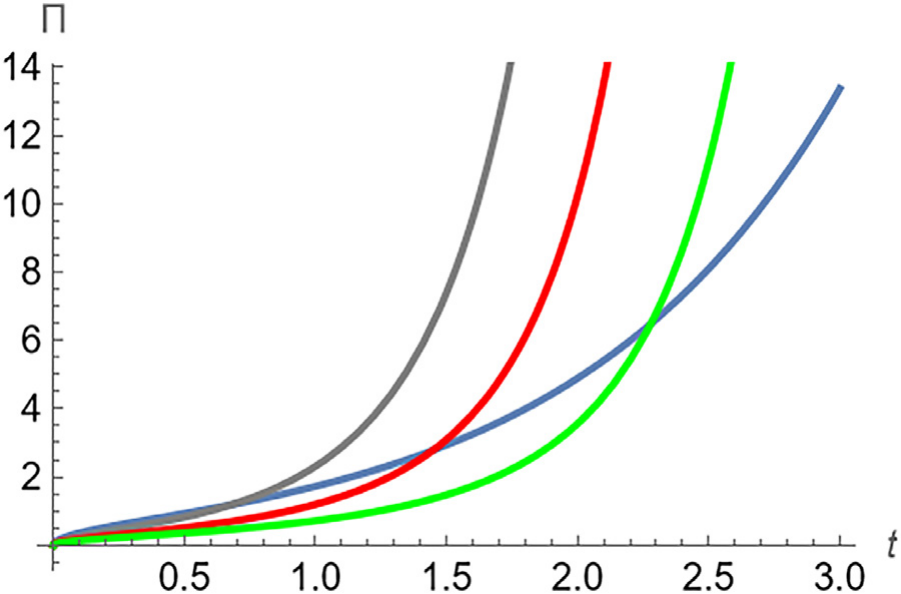
The plot of ${\left[\Pi_{0.5}\right]}_{1}\left(t^{0.5}\right)$ (blue), ${\left[\Pi_{0.5}\right]}_{2}\left(2t^{0.5}\right)$ (gray), ${\left[\Pi_{0.5}\right]}_{3}\left(2t^{0.5}\right)$ (red) and ${\left[\Pi_{0.5}\right]}_{4}\left(2t^{0.5}\right)$ (green) $\text{Plot}[{\left(\frac{t}{b}\right)}^{\frac{\frac{\kappa }{b}+1}{b}-1}*\text{MittagLefflerE}\left[\frac{1+\frac{\kappa }{b}}{b},\frac{1+\frac{\kappa }{b}}{b},\left(\frac{\lambda t^{\frac{1+\frac{\kappa }{b}}{b}}}{b^{\frac{1+\frac{\kappa }{b}}{b}}}\right)\right],\left\{t,0,3\right\}].$

A generalization of the Rabotnov function (see [8]) can be viewed by assuming $\Gamma_{b},$ as follows:

$$\begin{array}{ccc} {\left[\Pi_{\kappa }\right]}_{b}\left(\lambda t^{\kappa /b}\right) & = & \sum_{n=0}^{\infty }\left(\frac{\lambda^{n}t^{\frac{\left(n+1\right)\left(\kappa /b+1\right)}{b}-1}}{\Gamma_{b}\left[\left(n+1\right)\left(\kappa /b+1\right)\right]}\right),\lambda ,\kappa \in R^{+},b>0\\ & = & \sum_{n=0}^{\infty }\left(\frac{\lambda^{n}t^{\frac{\left(n+1\right)\left(\kappa /b+1\right)}{b}-1}}{b^{\frac{\left(n+1\right)\left(\kappa /b+1\right)}{b}-1}\Gamma \left[\frac{\left(n+1\right)\left(\kappa /b+1\right)}{b}\right]}\right)\\ & = & {\left(\frac{t}{b}\right)}^{\left(\kappa /b+1\right)/b-1}\sum_{n=0}^{\infty }\left(\frac{{\left[\lambda {\left(t/b\right)}^{\frac{\kappa /b+1}{b}}\right]}^{n}}{\Gamma \left[\frac{n\left(\kappa /b+1\right)+\left(\kappa /b+1\right)}{b}\right]}\right)\\ & = & {\left(\frac{t}{b}\right)}^{\left(\kappa /b+1\right)/b-1}\Xi_{\left(\frac{1+\kappa /b}{b},\frac{1+\kappa /b}{b}\right)}\left(\frac{\lambda t^{\left(1+\kappa /b\right)/b}}{b^{\left(1+\kappa /b\right)/b}}\right), \end{array}$$

where Ξ(.) is the Mittag-Leffler function (see Fig. 1).

It is clear that, when b=1, we obtain the normal Rabotnov function.

*Definition 1.* Suppose that ϕ(t) is a differentiable function over (0, ℘), then the b−Caputo fractal-fractional derivative is given by:

$${}_{b}^{C}\Delta_{t}^{\kappa ,\nu }\phi \left(t\right)=\int_{0}^{t}\frac{d\phi \left(\tau \right)}{d\tau^{\frac{\nu }{b}}}{\left[\Pi_{\kappa }\right]}_{b}\left(-\lambda {\left(t-\tau \right)}^{\frac{\kappa }{b}}\right)d\tau .$$

In addition, for a continuous function ϕ(t) and fractal differentiable over (0, ℘), the b−Riemann-Liouville fractal-fractional differential operator is designed by

$${}_{b}^{RL}\Delta_{t}^{\kappa ,\nu }\phi \left(t\right)=\frac{d}{dt^{\frac{\nu }{b}}}\int_{0}^{t}\phi \left(\tau \right){\left[\Pi_{\kappa }\right]}_{b}\left(-\lambda {\left(t-\tau \right)}^{\frac{\kappa }{b}}\right)d\tau$$

where

$$\frac{d\phi \left(t\right)}{dt^{\nu /b}}=\underset{t\to \tau }{\text{lim}}\frac{\phi \left(t\right)-\phi \left(\tau \right)}{t^{\nu /b}-\tau^{\nu /b}}.$$

Clearly, when b=1,ν=1, the above operators reduce into the normal formula [9,10]. Also, a generalization is given to the above operators, as follows:

$${}_{b}^{C}\Delta_{t}^{\kappa ,\nu ,\gamma }\phi \left(t\right)=\int_{0}^{t}\frac{d^{\gamma /b}\phi \left(\tau \right)}{d\tau^{\nu /b}}{\left[\Pi_{\kappa }\right]}_{b}\left(-\lambda {\left(t-\tau \right)}^{\frac{\kappa }{b}}\right)d\tau .$$

And

$${}_{b}^{RL}\Delta_{t}^{\kappa ,\nu^{,}\gamma }\phi \left(t\right)=\frac{d^{\gamma /b}}{dt^{\nu /b}}\int_{0}^{t}\phi \left(\tau \right){\left[\Pi_{\kappa }\right]}_{b}\left(-\lambda {\left(t-\tau \right)}^{\frac{\kappa }{b}}\right)d\tau ,$$

where

$$\frac{d^{\gamma /b}\phi \left(t\right)}{dt^{\nu /b}}=\underset{t\to \tau }{\text{lim}}\frac{\phi^{\gamma /b}\left(t\right)-\phi^{\gamma /b}\left(\tau \right)}{t^{\nu /b}-\tau^{\nu /b}}.$$

In an analogy, the b−fractal-fractional integral operator of order κ,ν>0 is given by

$${}_{b}J_{t}^{\kappa ,\nu }\phi \left(t\right)≔\nu \int_{0}^{t}\phi \left(\tau \right){\left[\Pi_{\kappa }\right]}_{b}(-\lambda {\left(t-\tau \right)}^{\frac{\kappa }{b}}d\tau .$$

Note that when b=1,ν=1 and γ=1 the above operators reduce into

*Example 1.* Let $\phi \left(t\right)=t^{\sigma },\sigma \in R.$ Since

$${\left[\Pi_{\kappa }\right]}_{b}\left(-\lambda {\left(t-\tau \right)}^{\kappa /b}\right)={\left(\frac{\left(t-\tau \right)}{b}\right)}^{\left(\kappa /b+1\right)/b-1}\Xi_{\left(\frac{1+\kappa /b}{b},\frac{1+\kappa /b}{b}\right)}\left(\frac{-\lambda {\left(t-\tau \right)}^{\left(1+\kappa /b\right)/b}}{b^{\left(1+\kappa /b\right)/b}}\right)$$

and

$$\frac{d\left(t^{\sigma }\right)}{dt^{\nu /b}}=\underset{t\to \tau }{\text{lim}}\frac{\left(t^{\sigma }\right)-\left(\tau^{\sigma }\right)}{t^{\nu /b}-\tau^{\nu /b}}=\frac{b\left(\sigma +1-\kappa \right)\tau^{1-\nu /b-\kappa +\sigma }}{\nu }$$

then, for c≔2−ν/b−κ+σ>0, β≔(κ/b+1)/b>0, we get

$$\begin{array}{ccc} & & {}_{b}^{C}\Delta_{t}^{\kappa ,\nu }\left(t^{\sigma }\right)\\ & & =\int_{0}^{t}\frac{d\left(\tau^{\sigma }\right)}{d\tau^{\nu /b}}{\left(\frac{\left(t-\tau \right)}{b}\right)}^{\left(\kappa /b+1\right)/b-1}\Xi_{\left(\frac{1+\kappa /b}{b},\frac{1+\kappa /b}{b}\right)}\left(\frac{-\lambda {\left(t-\tau \right)}^{\left(1+\kappa /b\right)/b}}{b^{\left(1+\kappa /b\right)/b}}\right)d\tau \\ & & =\left(\frac{b^{1-\beta }\left(\sigma +1-\kappa \right)}{\nu }\right)\int_{0}^{t}\tau^{c-1}{\left(t-\tau \right)}^{\beta -1}\Xi_{\left(\beta ,\beta \right)}\left(\frac{-\lambda {\left(t-\tau \right)}^{\beta }}{b^{\beta }}\right)d\tau \\ & & =\left(\frac{b^{1-\beta }\left(\sigma +1-\kappa \right)}{\nu }\right)\left(\Xi_{\left(\beta ,\beta +c\right)}\left(\frac{-\lambda {\left(t\right)}^{\beta }}{b^{\beta }}\right)t^{c+\beta -1}\right). \end{array}$$

By using the definition of ${}_{b}^{RL}\Delta_{t}^{\kappa ,\nu }$ together with β≔(κ/b+1)/b>0, σ≔ς−1>0 and ρ≔ς+β−1, we obtain

$$\begin{array}{ccc} & & {\;}_{b}^{RL}\Delta_{t}^{\kappa ,\nu }\left(t^{\sigma }\right)\\ & & =\frac{d}{dt^{\frac{\nu }{b}}}\int_{0}^{t}\left(\tau^{\sigma }\right){\left(\frac{\left(t-\tau \right)}{b}\right)}^{\left(\kappa /b+1\right)/b-1}\Xi_{\left(\frac{1+\kappa /b}{b},\frac{1+\kappa /b}{b}\right)}\left(\frac{-\lambda {\left(t-\tau \right)}^{\left(1+\kappa /b\right)/b}}{b^{\left(1+\kappa /b\right)/b}}\right)d\tau \\ & & =\frac{1}{b^{\beta -1}}\frac{d}{dt^{\frac{\nu }{b}}}\int_{0}^{t}\left(\tau^{\varsigma -1}\right){\left(t-\tau \right)}^{\beta -1}\Xi_{\left(\beta ,\beta \right)}\left(\frac{-\lambda {\left(t-\tau \right)}^{\beta }}{b^{\beta }}\right)d\tau \\ & & =\frac{1}{b^{\beta -1}}\frac{d}{dt^{\frac{\nu }{b}}}\left(t^{\varsigma +\beta -1}\Xi_{\left(\beta ,\beta +\varsigma \right)}\left(\frac{-\lambda {\left(t\right)}^{\beta }}{b^{\beta }}\right)\right)\\ & & =\frac{1}{\nu }{\left(\frac{1}{b}\right)}^{\beta -1}\left(bt^{\rho -\frac{\nu }{b}}(\rho \Xi_{\beta ,\beta +\varsigma }\left(-\lambda {\left(\frac{t}{b}\right)}^{\beta }\right)-\lambda {\left(\frac{t}{b}\right)}^{\beta }\left(\Xi_{\beta ,2\beta +\varsigma -1}\left(-\lambda {\left(\frac{t}{b}\right)}^{\beta }\right)-\rho \Xi_{\beta ,2\beta +\varsigma }\left(-\lambda {\left(\frac{t}{b}\right)}^{\beta }\right)\right)\right) \end{array}$$

Using the fractal-fractional integral operator, with the assumptionsβ≔(κ/b+1)/b>0, σ≔ς−1>0, we get

$$\begin{array}{ccc} & & {}_{b}J_{t}^{\kappa ,\nu }\left(t^{\sigma }\right)\\ & & =\nu \int_{0}^{t}\left(\tau^{\sigma }\right){\left[\Pi_{\kappa }\right]}_{b}(-\lambda {\left(t-\tau \right)}^{\frac{\kappa }{b}}d\tau \\ & & =\nu {\left(\frac{1}{b}\right)}^{\left(\kappa /b+1\right)/b-1}\int_{0}^{t}\left(\tau^{\sigma }\right){\left(t-\tau \right)}^{\left(\kappa /b+1\right)/b-1}\Xi_{\left(\frac{1+\kappa /b}{b},\frac{1+\kappa /b}{b}\right)}\left(\frac{-\lambda {\left(t-\tau \right)}^{\left(1+\kappa /b\right)/b}}{b^{\left(1+\kappa /b\right)/b}}\right)d\tau \\ & & =\nu {\left(\frac{1}{b}\right)}^{\left(\kappa /b+1\right)/b-1}\int_{0}^{t}\left(\tau^{\varsigma -1}\right){\left(t-\tau \right)}^{\beta -1}\Xi_{\left(\beta ,\beta \right)}\left(\frac{-\lambda {\left(t-\tau \right)}^{\beta }}{b^{\beta }}\right)d\tau \\ & & =\nu {\left(\frac{1}{b}\right)}^{\left(\kappa /b+1\right)/b-1}\Xi_{\left(\beta ,\beta +\varsigma \right)}\left(\frac{-\lambda {\left(t\right)}^{\beta }}{b^{\beta }}\right)t^{\beta +\varsigma -1}. \end{array}$$

Now, for the triple fractal-fractional order operators, we get

$$\begin{array}{ccc} \frac{d^{\gamma /b}\left(t^{\sigma }\right)}{dt^{\nu /b}} & = & \underset{t\to \tau }{\text{lim}}\frac{{\left(t^{\sigma }\right)}^{\gamma /b}-{\left(\tau^{\sigma }\right)}^{\gamma /b}}{t^{\nu /b}-\tau^{\nu /b}}\\ & = & \underset{t\to \tau }{\text{lim}}\frac{{\left(t\right)}^{\sigma \gamma /b}-{\left(\tau \right)}^{\sigma \gamma /b}}{t^{\nu /b}-\tau^{\nu /b}}=\frac{\sigma \gamma \;\tau^{\frac{\sigma \gamma -\nu }{b}}}{\nu }. \end{array}$$

Then, by letting β≔(1+κ/b)/b>0 and $\eta ≔\frac{\sigma \gamma -\nu }{b}+1>0$, we have

$$\begin{array}{ccc} {}_{b}^{C}\Delta_{t}^{\kappa ,\nu ,\gamma }\left(t^{\sigma }\right) & = & \int_{0}^{t}\frac{\sigma \gamma \;\tau^{\frac{\sigma \gamma -\nu }{b}}}{\nu }{\left(\frac{\left(t-\tau \right)}{b}\right)}^{\left(\kappa /b+1\right)/b-1}\Xi_{\left(\frac{1+\kappa /b}{b},\frac{1+\kappa /b}{b}\right)}\left(\frac{-\lambda {\left(t-\tau \right)}^{\left(1+\kappa /b\right)/b}}{b^{\left(1+\kappa /b\right)/b}}\right)d\tau \\ & = & \left(\frac{\sigma \gamma \;}{\nu }{\left(\frac{1}{b}\right)}^{\beta -1}\right)\int_{0}^{t}\tau^{\eta -1}{\left(t-\tau \right)}^{\beta -1}\Xi_{\left(\beta ,\beta \right)}\left(\frac{-\lambda {\left(t-\tau \right)}^{\beta }}{b^{\beta }}\right)d\tau \\ & = & \left(\frac{\sigma \gamma \;}{\nu }{\left(\frac{1}{b}\right)}^{\beta -1}\right)\int_{0}^{t}\tau^{\eta -1}{\left(t-\tau \right)}^{\beta -1}\Xi_{\left(\beta ,\beta \right)}\left(\frac{-\lambda {\left(t-\tau \right)}^{\beta }}{b^{\beta }}\right)d\tau \\ & = & \left(\frac{\sigma \gamma \;}{\nu }{\left(\frac{1}{b}\right)}^{\beta -1}\right)t^{\eta +\beta -1}\Xi_{\left(\beta ,\beta +\eta \right)}\left(\frac{-\lambda {\left(t\right)}^{\beta }}{b^{\beta }}\right). \end{array}$$

Moreover, for σ≔ς−1 and ρ≔ς+β−1, we have

$$\begin{array}{ccc} {}_{b}^{RL}\Delta_{t}^{\kappa ,\nu^{,}\gamma }\left(t^{\sigma }\right) & = & \frac{d^{\gamma /b}}{dt^{\nu /b}}\int_{0}^{t}\left(\tau^{\sigma }\right){\left(\frac{\left(t-\tau \right)}{b}\right)}^{\left(\kappa /b+1\right)/b-1}\Xi_{\left(\frac{1+\kappa /b}{b},\frac{1+\kappa /b}{b}\right)}\left(\frac{-\lambda {\left(t-\tau \right)}^{\left(1+\kappa /b\right)/b}}{b^{\left(1+\kappa /b\right)/b}}\right)d\tau \\ & = & {\left(\frac{1}{b}\right)}^{\beta -1}\frac{d^{\gamma /b}}{dt^{\nu /b}}\int_{0}^{t}\left(\tau^{\varsigma -1}\right){\left(t-\tau \right)}^{\beta -1}\Xi_{\left(\beta ,\beta \right)}\left(\frac{-\lambda {\left(t-\tau \right)}^{\beta }}{b^{\beta }}\right)d\tau \\ & = & {\left(\frac{1}{b}\right)}^{\beta -1}\frac{d^{\gamma /b}}{dt^{\nu /b}}t^{\varsigma +\beta -1}\Xi_{\left(\beta ,\beta +\varsigma \right)}\left(\frac{-\lambda {\left(t\right)}^{\beta }}{b^{\beta }}\right)\\ & = & \frac{t^{\frac{\gamma \rho -\nu }{b}}}{b^{2\beta -1}\nu }(\gamma \rho b^{\beta }\Xi_{\beta ,\beta +\varsigma }\left(-b^{-\beta -1}\lambda \gamma t^{\beta }\right)-\lambda \gamma t^{\beta }\left(\Xi_{\beta ,2\beta +\varsigma -1}\left(-b^{-\beta -1}\lambda \gamma t^{\beta }\right)-\rho \Xi_{\beta ,2\beta +\varsigma }\left(-b^{-\beta -1}\lambda \gamma t^{\beta }\right)\right)) \end{array}$$

where

$$\begin{array}{ccc} \frac{d^{\gamma /b}\phi \left(t\right)}{dt^{\nu /b}} & = & \underset{t\to \tau }{\text{lim}}\frac{{\left(t^{\rho }\Xi_{\left(\beta ,\beta +\varsigma \right)}\left(\frac{-\lambda {\left(t\right)}^{\beta }}{b^{\beta }}\right)\right)}^{\gamma /b}-{\left(\tau^{\rho }\Xi_{\left(\beta ,\beta +\varsigma \right)}\left(\frac{-\lambda {\left(\tau \right)}^{\beta }}{b^{\beta }}\right)\right)}^{\gamma /b}}{t^{\nu /b}-\tau^{\nu /b}}\\ & = & \underset{t\to \tau }{\text{lim}}\frac{\left(t^{\rho \gamma /b}\Xi_{\left(\beta ,\beta +\varsigma \right)}\left(\frac{-\lambda \gamma {\left(t\right)}^{\beta }}{b^{\beta +1}}\right)\right)-\left(\tau^{\rho \gamma /b}\Xi_{\left(\beta ,\beta +\varsigma \right)}\left(\frac{-\lambda \gamma {\left(\tau \right)}^{\beta }}{b^{\beta +1}}\right)\right)}{t^{\nu /b}-\tau^{\nu /b}}\\ & = & \frac{b^{-\beta }\tau^{\frac{\gamma \rho -\nu }{b}}}{\nu }\left(\gamma \rho b^{\beta }\Xi_{\beta ,\beta +\varsigma }\left(-b^{-\beta -1}\lambda \gamma \tau^{\beta }\right)-\lambda \gamma \tau^{\beta }\left(\Xi_{\beta ,2\beta +\varsigma -1}\left(-b^{-\beta -1}\lambda \gamma \tau^{\beta }\right)-\rho \Xi_{\beta ,2\beta +\varsigma }\left(-b^{-\beta -1}\lambda \gamma \tau^{\beta }\right)\right)\right) \end{array}$$

Next, we investigate the boundedness of the suggested operators. There are many advantages for checking the boundedness of fractal operators in general and fractal-fractional operators in particular. In particular in the areas of signal processing, image analysis, and data compression, bounded fractal operators can have a number of advantages. Images or signals can be represented at many resolutions using bounded fractal operators. This implies that they are able to collect data at various sizes, which makes it possible to analyze difficult patterns in the data in more depth. It is well known that fractals may depict complex designs using rather straightforward mathematical methods. Data may be effectively compressed using bounded fractal operators, which lowers storage needs while keeping key elements of the original data (see [9,10]).

*Proposition 1.* Consider the b−fractal-fractional differential operators, where ϕ is a continuous function on J=[0,℘] with $∥\phi ∥=\text{ma}x_{t\in J}\left|\phi (t)\right|.$ If λ≥0 and κ≠b then

• $|{}_{b}^{C}\Delta_{t}^{\kappa ,\nu }\phi \left(t\right)|\leq C_{1}∥\phi ∥,$ where $C_{1}≔\left(\frac{2b^{2}}{b+\kappa }{℘}^{\frac{b+\kappa }{b^{2}}-\left(\nu +\kappa /b+1\right)/b+1}\right).$
• $|{}_{b}^{RL}\Delta_{t}^{\kappa ,\nu }\phi \left(t\right)|\leq C_{2}∥\phi ∥,$ where $C_{2}≔\left(\frac{b^{3}{\left(\frac{1}{b}\right)}^{\frac{b+\kappa }{b^{2}}}}{b+\kappa }\right)\left(\frac{\left(\left(b+\kappa \right){℘}^{\kappa /b^{2}+\left(1-\nu \right)/b}\right)}{\left(b\nu \right)}\right).$
• $|{}_{b}J_{t}^{\kappa ,\nu }\phi \left(t\right)|\leq C_{3}∥\phi ∥,$ where $C_{3}≔\nu {\left(\frac{1}{b}\right)}^{(\kappa /b+1)/b-1}\frac{b^{2}{℘}^{\frac{b+\kappa }{b^{2}}}}{b+\kappa }.$

Obviously, from the definition of the fractal-fractional derivative, we have

$$\begin{array}{ccc} \left|{}_{b}^{C}\Delta_{t}^{\kappa ,\nu }\phi \left(t\right)\right| & = & \left|\int_{0}^{t}\frac{d\phi \left(\tau \right)}{d\tau^{\frac{\nu }{b}}}{\left[\Pi_{\kappa }\right]}_{b}\left(-\lambda {\left(t-\tau \right)}^{\frac{\kappa }{b}}\right)d\tau \right|\\ & \leq & \frac{2∥\phi ∥}{{℘}^{\frac{\nu }{b}}}\int_{0}^{t}\left|{\left(\frac{\left(t-\tau \right)}{b}\right)}^{\left(\kappa /b+1\right)/b-1}\Xi_{\left(\frac{1+\kappa /b}{b},\frac{1+\kappa /b}{b}\right)}\left(\frac{-\lambda {\left(t-\tau \right)}^{\left(1+\kappa /b\right)/b}}{b^{\left(1+\kappa /b\right)/b}}\right)\right|d\tau \\ & \leq & \frac{2∥\phi ∥}{{℘}^{\left(\nu +\kappa /b+1\right)/b-1}}\int_{0}^{t}\left|{\left(t-\tau \right)}^{\left(\kappa /b+1\right)/b-1}\right|d\tau \\ & = & \frac{2∥\phi ∥}{{℘}^{\left(\nu +\kappa /b+1\right)/b-1}}\frac{b^{2}t^{\frac{b+\kappa }{b^{2}}}}{b+\kappa }\\ & \leq & \left(\frac{2b^{2}}{b+\kappa }{℘}^{\frac{b+\kappa }{b^{2}}-\left(\nu +\kappa /b+1\right)/b+1}\right)∥\phi ∥\\ & & ≔C_{1}∥\phi ∥. \end{array}$$

For the second operator, we get

$$\begin{array}{ccc} \left|{}_{b}^{RL}\Delta_{t}^{\kappa ,\nu }\phi \left(t\right)\right| & = & \left|\frac{d}{dt^{\nu /b}}\int_{0}^{t}\phi \left(\tau \right){\left(\frac{\left(t-\tau \right)}{b}\right)}^{\left(\kappa /b+1\right)/b-1}\Xi_{\left(\frac{1+\kappa /b}{b},\frac{1+\kappa /b}{b}\right)}\left(\frac{-\lambda {\left(t-\tau \right)}^{\left(1+\kappa /b\right)/b}}{b^{\left(1+\kappa /b\right)/b}}\right)d\tau \right|\\ & \leq & ∥\phi ∥\frac{d}{dt^{\nu /b}}\int_{0}^{t}\left|{\left(\frac{\left(t-\tau \right)}{b}\right)}^{\left(\kappa /b+1\right)/b-1}\Xi_{\left(\frac{1+\kappa /b}{b},\frac{1+\kappa /b}{b}\right)}\left(\frac{-\lambda {\left(t-\tau \right)}^{\left(1+\kappa /b\right)/b}}{b^{\left(1+\kappa /b\right)/b}}\right)\right|d\tau \\ & \leq & ∥\phi ∥\frac{d}{dt^{\nu /b}}\int_{0}^{t}\left|{\left(\frac{\left(t-\tau \right)}{b}\right)}^{\left(\kappa /b+1\right)/b-1}\right|d\tau \\ & = & ∥\phi ∥\frac{d}{dt^{\nu /b}}\left(\frac{b^{3}{\left(\frac{t}{b}\right)}^{\frac{b+\kappa }{b^{2}}}}{b+\kappa }\right)\\ & = & ∥\phi ∥\left(\frac{b^{3}{\left(\frac{1}{b}\right)}^{\frac{b+\kappa }{b^{2}}}}{b+\kappa }\right)\left(\frac{\left(\left(b+\kappa \right)t^{\kappa /b^{2}+\left(1-\nu \right)/b}\right)}{\left(b\nu \right)}\right)\\ & \leq & ∥\phi ∥\left(\frac{b^{3}{\left(\frac{1}{b}\right)}^{\frac{b+\kappa }{b^{2}}}}{b+\kappa }\right)\left(\frac{\left(\left(b+\kappa \right){℘}^{\kappa /b^{2}+\left(1-\nu \right)/b}\right)}{\left(b\nu \right)}\right)\\ & = & C_{2}∥\phi ∥. \end{array}$$

For the integral operator, we obtain

$$\begin{array}{ccc} \left|{}_{b}J_{t}^{\kappa ,\nu }\phi \left(t\right)\right| & \leq & \nu \int_{0}^{t}\left|\phi \left(\tau \right)\right|\left|{\left[\Pi_{\kappa }\right]}_{b}(-\lambda {\left(t-\tau \right)}^{\frac{\kappa }{b}}\right|d\tau \\ & \leq & ∥\phi ∥\nu \int_{0}^{t}\left|{\left(\frac{\left(t-\tau \right)}{b}\right)}^{\left(\kappa /b+1\right)/b-1}\Xi_{\left(\frac{1+\kappa /b}{b},\frac{1+\kappa /b}{b}\right)}\left(\frac{-\lambda {\left(t-\tau \right)}^{\left(1+\kappa /b\right)/b}}{b^{\left(1+\kappa /b\right)/b}}\right)\right|d\tau \\ & \leq & ∥\phi ∥\nu {\left(\frac{1}{b}\right)}^{\left(\kappa /b+1\right)/b-1}\int_{0}^{t}\left|{\left(t-\tau )\right)}^{\left(\kappa /b+1\right)/b-1}\right|d\tau \\ & \leq & ∥\phi ∥\nu {\left(\frac{1}{b}\right)}^{\left(\kappa /b+1\right)/b-1}\frac{b^{2}t^{\frac{b+\kappa }{b^{2}}}}{b+\kappa }\\ & \leq & ∥\phi ∥\nu {\left(\frac{1}{b}\right)}^{\left(\kappa /b+1\right)/b-1}\frac{b^{2}{℘}^{\frac{b+\kappa }{b^{2}}}}{b+\kappa }\\ ≔C_{3}∥\phi ∥. \end{array}$$

## Method validation

The particular circumstances of the equations and the corresponding theorems determine the existence and uniqueness of solutions to differential equations. The standards for determining these attributes may vary throughout differential equation categories. In order to analyze and solve differential equations, the use of theorems such as the Picard-Lindelof theorem and uniqueness theorem is essential. In this section, we consider the following abstract fractal-fractional differential equation

(1) $${}_{b}^{C}\Delta_{t}^{\kappa ,\nu }\upsilon \left(t\right)=V\left(t,\upsilon \left(t\right)\right);$$

or

(2) $${}_{b}^{RL}\Delta_{t}^{\kappa ,\nu }\upsilon \left(t\right)=V\left(t,\upsilon \left(t\right)\right),$$

where $\upsilon \left(0\right)=\upsilon_{0}$ and V:[0,℘]×R→R is continuous and differentiable. Consider the Banach space B of all continuous functions over the interval J=[0,℘],℘<∞ subjecting to the sup norm.

Next result shows the existence of nonzero solution for Eq. (1).

*Theorem 1.* Consider the fractal-fractional equation ((1) or (2)). If V is bounded in its domain with ∥V∥≤L∥υ∥,∥υ∥>0,L∈[0,∞) then Eq. (1) admits a solution υ. In addition, if V is a Lipschitz with respect to the second variable

∥V(t,υ(t))−V(t,ψ(t))∥≤l∥υ(t)−ψ(t)∥,

such that

$$l\langle \frac{1}{\nu {\left(\frac{1}{b}\right)}^{\left(\kappa /b+1\right)/b-1}\frac{b^{2}{℘}^{\frac{b+\kappa }{b^{2}}}}{b+\kappa }},\nu \rangle 0$$

then the solution is unique.

*Proof.*

Define the following operator: Q:S→S, where

$$S≔\left\{\upsilon \in B:∥\upsilon ∥\leq \frac{2\left|\upsilon_{0}\right|}{1-L\nu {\left(\frac{1}{b}\right)}^{\left(\kappa /b+1\right)/b-1}\left(\frac{b^{2}{℘}^{\frac{b+\kappa }{b^{2}}}}{b+\kappa }\right)}\right\},$$

t∈[0,℘],℘<∞ and

$$L\nu {\left(\frac{1}{b}\right)}^{\left(\kappa /b+1\right)/b-1}\left(\frac{b^{2}{℘}^{\frac{b+\kappa }{b^{2}}}}{b+\kappa }\right)<1$$

by

$$\begin{array}{cc} Q\left(\upsilon \right)\left(t\right) & =\upsilon_{0}+\nu \int_{0}^{t}V\left(\tau ,\upsilon \left(\tau \right)\right){\left[\Pi_{\kappa }\right]}_{b}(-\lambda {\left(t-\tau \right)}^{\frac{\kappa }{b}}d\tau . \end{array}$$

Then we have

$$\begin{array}{c} \left|Q\left(\upsilon \right)\left(t\right)\right|=\left|\upsilon_{0}+\nu \int_{0}^{t}V\left(\tau ,\upsilon \left(\tau \right)\right){\left[\Pi_{\kappa }\right]}_{b}(-\lambda {\left(t-\tau \right)}^{\frac{\kappa }{b}}d\tau \right|\\ \leq \left|\upsilon_{0}\right|+\nu \left|\int_{0}^{t}V\left(\tau ,\upsilon \left(\tau \right)\right){\left[\Pi_{\kappa }\right]}_{b}(-\lambda {\left(t-\tau \right)}^{\frac{\kappa }{b}}d\tau \right|\\ \leq \left|\upsilon_{0}\right|+\nu \int_{0}^{t}\left|V\left(\tau ,\upsilon \left(\tau \right)\right)\right|\left|{\left[\Pi_{\kappa }\right]}_{b}(-\lambda {\left(t-\tau \right)}^{\frac{\kappa }{b}}\right|d\tau \\ \leq \left|\upsilon_{0}\right|+\nu L∥\upsilon ∥\int_{0}^{t}\left|{\left(\frac{\left(t-\tau \right)}{b}\right)}^{\left(\kappa /b+1\right)/b-1}\Xi_{\left(\frac{1+\kappa /b}{b},\frac{1+\kappa /b}{b}\right)}\left(\frac{-\lambda {\left(t-\tau \right)}^{\left(1+\kappa /b\right)/b}}{b^{\left(1+\kappa /b\right)/b}}\right)\right|d\tau \\ \leq \left|\upsilon_{0}\right|+\nu L∥\upsilon ∥{\left(\frac{1}{b}\right)}^{\left(\kappa /b+1\right)/b-1}\\ \times \int_{0}^{t}\left|{\left(t-\tau )\right)}^{\left(\kappa /b+1\right)/b-1}\Xi_{\left(\frac{1+\kappa /b}{b},\frac{1+\kappa /b}{b}\right)}\left(\frac{-\lambda {\left(t-\tau \right)}^{\left(1+\kappa /b\right)/b}}{b^{\left(1+\kappa /b\right)/b}}\right)\right|d\tau \\ \leq \left|\upsilon_{0}\right|+\nu L∥\upsilon ∥{\left(\frac{1}{b}\right)}^{\left(\kappa /b+1\right)/b-1}\int_{0}^{t}\left|{\left(t-\tau )\right)}^{\left(\kappa /b+1\right)/b-1}\right|d\tau \\ \leq \left|\upsilon_{0}\right|+\nu L∥\upsilon ∥{\left(\frac{1}{b}\right)}^{\left(\kappa /b+1\right)/b-1}\left(\frac{b^{2}{℘}^{\frac{b+\kappa }{b^{2}}}}{b+\kappa }\right) \end{array}$$

Thus, we obtain

$$\left|Q\left(\upsilon \right)\left(t\right)\right|\leq \frac{\left|\upsilon_{0}\right|}{1-L\nu {\left(\frac{1}{b}\right)}^{\left(\kappa /b+1\right)/b-1}\left(\frac{b^{2}{℘}^{\frac{b+\kappa }{b^{2}}}}{b+\kappa }\right)},\upsilon_{0}\neq 0.$$

By taking the supremum of the above inequality, we have Q∈S (boundedness of Q). We proceed to show that Q is continuous in S. Since V is continuous in J then it is uniformly continuous satisfying

$$∥V\left(t,\upsilon \left(t\right)\right)-V\left(t,\psi \left(t\right)\right)∥\leq \frac{\epsilon }{\nu {\left(\frac{1}{b}\right)}^{\left(\kappa /b+1\right)/b-1}\frac{b^{2}{℘}^{\frac{b+\kappa }{b^{2}}}}{b+\kappa }}.$$

A computation brings

$$\begin{array}{c} \left|Q\left(\upsilon \right)\left(t\right)-Q\left(\psi \right)\left(t\right)\right|\\ \leq \nu \int_{0}^{t}\left|V\left(\tau ,\upsilon \left(\tau \right)\right)-V\left(\tau ,\psi \left(\tau \right)\right)\right|\left|{\left[\Pi_{\kappa }\right]}_{b}(-\lambda {\left(t-\tau \right)}^{\frac{\kappa }{b}}\right|d\tau \\ \leq \nu ∥V\left(t,\upsilon \left(t\right)\right)-V\left(t,\psi \left(t\right)\right)∥\int_{0}^{t}\left|{\left(\frac{\left(t-\tau \right)}{b}\right)}^{\left(\kappa /b+1\right)/b-1}\Xi_{\left(\frac{1+\kappa /b}{b},\frac{1+\kappa /b}{b}\right)}\left(\frac{-\lambda {\left(t-\tau \right)}^{\left(1+\kappa /b\right)/b}}{b^{\left(1+\kappa /b\right)/b}}\right)\right|d\tau \\ \leq \nu ∥V\left(t,\upsilon \left(t\right)\right)-V\left(t,\psi \left(t\right)\right)∥{\left(\frac{1}{b}\right)}^{\left(\kappa /b+1\right)/b-1}\\ \times \int_{0}^{t}\left|{\left(t-\tau )\right)}^{\left(\kappa /b+1\right)/b-1}\Xi_{\left(\frac{1+\kappa /b}{b},\frac{1+\kappa /b}{b}\right)}\left(\frac{-\lambda {\left(t-\tau \right)}^{\left(1+\kappa /b\right)/b}}{b^{\left(1+\kappa /b\right)/b}}\right)\right|d\tau \\ \leq ∥V\left(t,\upsilon \left(t\right)\right)-V\left(t,\psi \left(t\right)\right)∥\nu {\left(\frac{1}{b}\right)}^{\left(\kappa /b+1\right)/b-1}\int_{0}^{t}\left|{\left(t-\tau )\right)}^{\left(\kappa /b+1\right)/b-1}\right|d\tau \\ \leq \frac{\varepsilon \nu }{\nu {\left(\frac{1}{b}\right)}^{\left(\kappa /b+1\right)/b-1}\frac{b^{2}{℘}^{\frac{b+\kappa }{b^{2}}}}{b+\kappa }}{\left(\frac{1}{b}\right)}^{\left(\kappa /b+1\right)/b-1}\frac{b^{2}{℘}^{\frac{b+\kappa }{b^{2}}}}{b+\kappa }\\ =\varepsilon . \end{array}$$

Let $t_{1},t_{2}\in J$ such that $t_{1}\neq t_{2}.$ Then

$$\begin{array}{c} \left|Q\left(\upsilon \right)\left(t_{1}\right)-Q\left(\upsilon \right)\left(t_{2}\right)\right|\\ \leq 2\left|\upsilon_{0}\right|+\nu \int_{0}^{t_{1}}\left|V\left(\tau ,\upsilon \left(\tau \right)\right)\right|\left|{\left[\Pi_{\kappa }\right]}_{b}(-\lambda {\left(t_{1}-\tau \right)}^{\frac{\kappa }{b}}\right|d\tau +\nu \int_{0}^{t_{2}}\left|V\left(\tau ,\upsilon \left(\tau \right)\right)\right|\left|{\left[\Pi_{\kappa }\right]}_{b}(-\lambda {\left(t_{2}-\tau \right)}^{\frac{\kappa }{b}}\right|d\tau \\ \leq 2\left|\upsilon_{0}\right|+L\nu ∥\upsilon ∥\int_{0}^{t_{1}}\left|{\left(\frac{\left(t_{1}-\tau \right)}{b}\right)}^{\left(\kappa /b+1\right)/b-1}\Xi_{\left(\frac{1+\kappa /b}{b},\frac{1+\kappa /b}{b}\right)}\left(\frac{-\lambda {\left(t_{1}-\tau \right)}^{\left(1+\kappa /b\right)/b}}{b^{\left(1+\kappa /b\right)/b}}\right)\right|d\tau \\ +L\nu ∥\upsilon ∥\int_{0}^{t_{2}}\left|{\left(\frac{\left(t_{2}-\tau \right)}{b}\right)}^{\left(\kappa /b+1\right)/b-1}\Xi_{\left(\frac{1+\kappa /b}{b},\frac{1+\kappa /b}{b}\right)}\left(\frac{-\lambda {\left(t_{2}-\tau \right)}^{\left(1+\kappa /b\right)/b}}{b^{\left(1+\kappa /b\right)/b}}\right)\right|d\tau \\ \leq 2\left|\upsilon_{0}\right|+\nu L∥\upsilon ∥{\left(\frac{1}{b}\right)}^{\left(\kappa /b+1\right)/b-1}\left(\int_{0}^{t_{1}}\left|{\left(t_{1}-\tau )\right)}^{\left(\kappa /b+1\right)/b-1}\right|d\tau +\int_{0}^{t_{2}}\left|{\left(t_{2}-\tau )\right)}^{\left(\kappa /b+1\right)/b-1}\right|d\tau \right)\\ \leq 2\left|\upsilon_{0}\right|+2\nu L∥\upsilon ∥{\left(\frac{1}{b}\right)}^{\frac{\frac{\kappa }{b}+1}{b}-1}\left(\frac{b^{2}{℘}^{\frac{b+\kappa }{b^{2}}}}{b+\kappa }\right) \end{array}$$

Thus, we obtain

$$\left|Q\left(\upsilon \right)\left(t_{1}\right)-Q\left(\upsilon \right)\left(t_{2}\right)\right|\leq \frac{2\left|\upsilon_{0}\right|}{1-L\nu {\left(\frac{1}{b}\right)}^{\left(\kappa /b+1\right)/b-1}\left(\frac{b^{2}{℘}^{\frac{b+\kappa }{b^{2}}}}{b+\kappa }\right)},\upsilon_{0}\neq 0.$$

Thus, Q is eqicontinuous in S. As a consequence, Arzela-Ascoli theorem yields that Q(S) is relatively compact operator. The Schauder fixed point theorem possesses a fixed point which is corresponding to the solution of (1).

For the uniqueness, we have to employ the Banach fixed point theorem.

$$\begin{array}{c} \left|Q\left(\upsilon \right)\left(t\right)-Q\left(\psi \right)\left(t\right)\right|\\ \leq \nu \int_{0}^{t}\left|V\left(\tau ,\upsilon \left(\tau \right)\right)-V\left(\tau ,\psi \left(\tau \right)\right)\right|\left|{\left[\Pi_{\kappa }\right]}_{b}(-\lambda {\left(t-\tau \right)}^{\frac{\kappa }{b}}\right|d\tau \\ \leq ∥V\left(t,\upsilon \left(t\right)\right)-V\left(t,\psi \left(t\right)\right)∥\nu \int_{0}^{t}\left|{\left(\frac{\left(t-\tau \right)}{b}\right)}^{\left(\kappa /b+1\right)/b-1}\Xi_{\left(\frac{1+\kappa /b}{b},\frac{1+\kappa /b}{b}\right)}\left(\frac{-\lambda {\left(t-\tau \right)}^{\left(1+\kappa /b\right)/b}}{b^{\left(1+\kappa /b\right)/b}}\right)\right|d\tau \\ \leq ∥V\left(t,\upsilon \left(t\right)\right)-V\left(t,\psi \left(t\right)\right)∥\nu {\left(\frac{1}{b}\right)}^{\left(\kappa /b+1\right)/b-1}\\ \times \int_{0}^{t}\left|{\left(t-\tau )\right)}^{\left(\kappa /b+1\right)/b-1}\Xi_{\left(\frac{1+\kappa /b}{b},\frac{1+\kappa /b}{b}\right)}\left(\frac{-\lambda {\left(t-\tau \right)}^{\left(1+\kappa /b\right)/b}}{b^{\left(1+\kappa /b\right)/b}}\right)\right|d\tau \\ \leq ∥V\left(t,\upsilon \left(t\right)\right)-V\left(t,\psi \left(t\right)\right)∥\nu {\left(\frac{1}{b}\right)}^{\left(\kappa /b+1\right)/b-1}\int_{0}^{t}\left|{\left(t-\tau )\right)}^{\left(\kappa /b+1\right)/b-1}\right|d\tau \\ \leq \left(\ell \nu {\left(\frac{1}{b}\right)}^{\left(\kappa /b+1\right)/b-1}\frac{b^{2}{℘}^{\frac{b+\kappa }{b^{2}}}}{b+\kappa }\right)∥\upsilon -\psi ∥, \end{array}$$

where

$$\left(l\nu {\left(\frac{1}{b}\right)}^{\left(\kappa /b+1\right)/b-1}\frac{b^{2}{℘}^{\frac{b+\kappa }{b^{2}}}}{b+\kappa }\right)<1.$$

Hence, the operator Q is a contraction mapping, then the Banach fixed point theorem shows that Q has a single fixed point that relates to the outcome of Eq. (1).

*Example 2.* Consider the following equation

(3) $${}_{1}^{C}\Delta_{t}^{1/2,1/2}\upsilon \left(t\right)=\frac{1}{8}\upsilon \left(t\right);t\in \left[0,1\right],$$

subjected initially to $\upsilon_{0}=1/2$ with l=L=1/8 and ν=κ=1/2 and ℘=b=1. Since,

$$\begin{array}{cc} L\nu {\left(\frac{1}{b}\right)}^{\left(\kappa /b+1\right)/b-1}\left(\frac{b^{2}{℘}^{\frac{b+\kappa }{b^{2}}}}{b+\kappa }\right) & =\frac{1}{8}\times \frac{1}{2}\times \frac{2}{3}=\frac{1}{24}<1. \end{array}$$

Thus, Eq. (3) has a solution in S. Moreover, it satisfies

$$\begin{array}{c} \frac{1}{\nu {\left(\frac{1}{b}\right)}^{\left(\kappa /b+1\right)/b-1}\frac{b^{2}{℘}^{\frac{b+\kappa }{b^{2}}}}{b+\kappa }}=\frac{4}{3}>l=\frac{1}{8}. \end{array}$$

Hence, it admits a unique solution with the formula

$$\begin{array}{cc} \upsilon \left(t\right) & =\upsilon_{0}+\nu \int_{0}^{t}V\left(\tau ,\upsilon \left(\tau \right)\right){\left[\Pi_{\kappa }\right]}_{b}(-\lambda {\left(t-\tau \right)}^{\frac{\kappa }{b}}d\tau \\ & =\frac{1}{2}+\frac{1}{2}\int_{0}^{t}\frac{\upsilon \left(\tau \right)}{8}{\left[\Pi_{\frac{1}{2}}\right]}_{1}(-\lambda {\left(t-\tau \right)}^{\frac{1}{2}}d\tau . \end{array}$$

Now, let b=2, then

$$\begin{array}{cc} L\nu {\left(\frac{1}{b}\right)}^{\left(\kappa /b+1\right)/b-1}\left(\frac{b^{2}{℘}^{\frac{b+\kappa }{b^{2}}}}{b+\kappa }\right) & =\frac{0.125{\left(\frac{1}{b}\right)}^{\left(b+0.5\right)b^{2}-3)}}{\left(b+0.5\right)}=0.12968<1. \end{array}$$

Thus, in view of Theorem 1, Eq. (3) has a solution. In addition, since

$$\begin{array}{c} \frac{1}{\nu {\left(\frac{1}{b}\right)}^{\left(\kappa /b+1\right)/b-1}\frac{b^{2}{℘}^{\frac{b+\kappa }{b^{2}}}}{b+\kappa }}=\frac{1}{{\left(\frac{1}{2}\right)}^{\left(\kappa /2+1\right)/2-1}\frac{2^{2}}{2+1/2}}=0.96388>l=0.125. \end{array}$$

Hence, it admits a unique solution. The solution takes the formula

$$\begin{array}{cc} \upsilon \left(t\right) & =\upsilon_{0}+\nu \int_{0}^{t}V\left(\tau ,\upsilon \left(\tau \right)\right){\left[\Pi_{\kappa }\right]}_{b}(-\lambda {\left(t-\tau \right)}^{\frac{\kappa }{b}}d\tau \\ & =\frac{1}{2}+\frac{1}{2}\int_{0}^{t}\frac{\upsilon \left(\tau \right)}{8}{\left[\Pi_{\frac{1}{2}}\right]}_{2}(-\lambda {\left(t-\tau \right)}^{\frac{1}{4}}d\tau . \end{array}$$

When b=3, we get

$$\begin{array}{cc} L\nu {\left(\frac{1}{b}\right)}^{\left(\kappa /b+1\right)/b-1}\left(\frac{b^{2}{℘}^{\frac{b+\kappa }{b^{2}}}}{b+\kappa }\right) & =0.314505<1. \end{array}$$

Therefore, by Theorem 1, Eq. (3) has a solution. Also, since

$$\begin{array}{c} \frac{1}{\nu {\left(\frac{1}{b}\right)}^{\left(\kappa /b+1\right)/b-1}\frac{b^{2}{℘}^{\frac{b+\kappa }{b^{2}}}}{b+\kappa }}=0.397449>l=0.125. \end{array}$$

Hence, it admits a unique solution. The solution takes the formula

$$\begin{array}{cc} \upsilon \left(t\right) & =\upsilon_{0}+\int_{0}^{t}V\left(\tau ,\upsilon \left(\tau \right)\right){\left[\Pi_{\kappa }\right]}_{b}(-\lambda {\left(t-\tau \right)}^{\frac{\kappa }{b}}d\tau \\ & =\frac{1}{2}+\frac{1}{2}\int_{0}^{t}\frac{\upsilon \left(\tau \right)}{8}{\left[\Pi_{\frac{1}{2}}\right]}_{3}(-\lambda {\left(t-\tau \right)}^{\frac{1}{6}}d\tau . \end{array}$$

***Remark 1*** Example 2 is a special type of Fractal-fractional Riccati equation. In several distinct domains, including differential geometry, mathematical physics, and control theory, the Riccati equation exists. It could sometimes be feasible to find closed-form solutions to the general Riccati problem, given its seemingly straightforward nature. The Riccati equation is commonly used in control theory to analyze optimum control issues, where finding the solution is crucial to identifying the best control tactics. It occurs in nonlinear differential equation problems in mathematical physics, and its solutions are frequently important for comprehending the movements of physical systems.

## Conclusions

A generalized set of different fractal-fractional operators, based on the modified b− Rabotnov function is given. Examples are the most challenge for these operators. Therefore, we illustrated different examples to show the behavior of the power function under these operators. These examples can be used to apply more interesting applications such as the special functions. The boundedness of the suggested b−fractal-fractional operators is investigated. In addition, we established sufficient conditions for the existence and uniqueness of solutions for abstract b−fractal-fractional differential equations. For future works, one can enhance the assumptions of Theorem 1 depending on the value ofb.

## Ethics authors statements

The platforms’ data redistribution policies were complied with.

## Funding statement

The paper is funded by Imam Mohammad Ibn Saud Islamic University (IMSIU), under the number IFP-IMSIU- 2023128.

## Data availability

No data was used for the research described in the article.

## CRediT authorship contribution statement

**Ibtehal Alazman:** Conceptualization, Methodology, Writing – original draft. **Rabha W. Ibrahim:** Visualization, Investigation, Software, Writing – original draft.

## Declaration of competing interest

The authors declare that they have no known competing financial interests or personal relationships that could have appeared to influence the work reported in this paper.

## Data Availability

No data was used for the research described in the article.
